# Prescription patterns of inhaler medications from 2017 to 2023: A retrospective study using Ontario administrative healthcare data

**DOI:** 10.1371/journal.pone.0348119

**Published:** 2026-06-10

**Authors:** Anthony Train, Krishna K. Vemuri, Angela Coderre-Ball, Javier Silva-Valencia, Declan Mulligan, Debra A. Butt, Jessica Gronsbell, Braden O’Neill, Andrea Gershon, Karen Tu

**Affiliations:** 1 Research and Innovation, North York General Hospital, Toronto, ON, Canada; 2 Department of Family Medicine, Queen’s University, Kingston, ON, Canada; 3 Department of Family and Community Medicine, Temerty Faculty of Medicine, University of Toronto, Toronto, ON, Canada; 4 Departments of Statistical Sciences, Family and Community Medicine, Computer Science, University of Toronto, Toronto, ON, Canada; 5 BC Psychosis Program, University of British Columbia Hospital, Vancouver Coastal Health, Vancouver, BC, Canada; 6 Department of Medicine, Sunnybrook Health Sciences Centre, Toronto, ON, Canada; 7 Department of Family and Community Medicine, Institute for Health Policy, Management and Evaluation, University of Toronto, Toronto, ON, Canada; College of Medical Sciences, NEPAL

## Abstract

**Background:**

Inhalers are essential for respiratory disease management, but commonly used metered-dose inhalers (MDIs) can often be replaced, when clinically appropriate, with lower-emission alternatives such as dry powder inhalers (DPIs) or soft mist inhalers (SMIs). The COVID-19 pandemic may have influenced prescribing patterns. We aim to describe changes in inhaler prescription trends and determine whether MDI use decreased during the pandemic.

**Methods and findings:**

Retrospective cohort study using Ontario’s health administrative data. Individuals aged 65 and older with new inhaler prescription instances from April 1, 2017, to March 31, 2023, were included. Monthly prescription event rates by inhaler type and socioeconomic factors were modeled for pre-pandemic (April 2017–March 2020) and pandemic (April 2020–March 2023) periods using interrupted time series and Generalized Additive Models to account for autocorrelation and seasonality. 750,517 new inhaler prescriptions were made during the study period. A significant drop was observed at the pandemic’s onset. MDIs were the predominant type of inhaler initially prescribed for both pre-pandemic ~77% and pandemic ~70% periods. The proportion of DPI initial prescriptions modestly increased from ~17% to ~23% and SMI initial prescriptions remained similar. Inhaler prescription rates did not significantly differ across sex, income quintiles, or rurality.

**Conclusions:**

The COVID-19 pandemic disrupted inhaler prescribing patterns in Ontario, leading to a temporary decline in new prescriptions. Despite environmental and guideline recommendations, MDIs continued to dominate prescribing practices. These findings underscore the need for targeted interventions to align clinical practices with current guidelines and environmental sustainability goals.

## Introduction

Inhalers, widely used for managing chronic respiratory diseases such as asthma and chronic obstructive pulmonary disease (COPD), are among the most prescribed medications globally. [1,2] In Canada, beta-agonist drugs like Salbutamol were ranked 9th among the top ten most prescribed medications in 2021. [3] However, traditional metered-dose inhalers (MDIs) pose an environmental challenge, as each device generates carbon emissions equivalent to driving 139 kilometers in a midsize vehicle [4] and has a greenhouse effect ten times greater than dry powder inhalers (DPIs), a readily available and more sustainable alternative.

Several Canadian healthcare bodies have called for changes in prescribing patterns to improve the environmental sustainability of the Canadian healthcare system. [4–8] Recommendations include reducing unnecessary inhaler prescriptions by improving diagnosis through spirometry and pulmonary function testing, [9] and switching from MDIs to less polluting alternatives, such as DPIs or soft mist inhalers (SMIs). [6] Little is known of how well these recommendations are followed, particularly in the context of recent disruptions to healthcare practices.

The COVID-19 pandemic likely influenced inhaler prescribing patterns in recent years. Infection control measures, such as social distancing and mask-wearing, significantly reduced the incidence of infectious diseases and respiratory illnesses. [10] It also led to a sharp decline in outpatient visits, with some regions reporting decreases of up to 73%. [11,12] The reductions in respiratory illnesses and associated outpatient visits likely decreased prescriptions of some inhalers. However, in the early stages of the pandemic, certain inhalers were being investigated as a potential treatment for COVID-19. [13–15] Consequently, inhalers, which were frequently prescribed off-label for general respiratory symptoms, may have seen increased use despite the limited evidence supporting their effectiveness for these indications. [16]

Given the substantial carbon footprint of MDIs, understanding their prescribing patterns is essential to inform policies and clinical strategies that support lower-emission inhaler use when clinically appropriate. Previous studies of prescription patterns have mainly focused on the carbon impact of these devices. [17–19] Comprehensive studies on Canadian inhaler prescribing patterns in the context of increased awareness of carbon footprints are lacking. This study aims to analyze prescribing patterns in Ontario from 2017 to 2023 among patients aged 65 and older, assessing whether prescriptions for high-carbon-impact MDIs decreased in favor of more environmentally sustainable inhalers, such as DPIs and SMIs, while accounting for the impact of the COVID-19 pandemic on prescribing trends.

## Methods

We conducted a retrospective longitudinal cohort study using health administrative data from Ontario, Canada, to examine changes in new inhaler prescriptions from 2017 to 2023. The analysis focused on overall prescription trends, variations by inhaler type, and differences across socioeconomic characteristics. This study was approved by the Research Ethics Board of North York General Hospital (# 0256).

### Data sources

We utilized three health administrative databases for Ontario, Canada. The Ontario Health Insurance Program (OHIP) collects billing information from physicians and hospital services. The Ontario Drug Benefit (ODB) database provides prescription medications dispensed from Ontario pharmacies, covering Ontarians aged 65 and older, social assistance recipients, and Trillium drug program members. The Registered Persons Database (RPDB) includes demographic data such as age and sex for all residents. Data for this study were accessed through the Ontario Health Data Platform, a Government of Ontario initiative, from November 2023 to July 2024. This study followed the Strengthening the Reporting of Observational Studies in Epidemiology Statement [20] and the REporting of studies Conducted using Observational Routinely-collected health Data (RECORD) statement. [21]

### Study population

The study cohort comprised Ontario residents aged 65 and older with at least one healthcare contact between April 1, 2017, and March 31, 2023. Individuals were followed until they turned 105, were lost to follow-up, died, or until the study ended. We included those newly prescribed an inhaler during the study period, defined as the first prescription with no other inhaler prescription in the previous 365 days, across subgroups by age, sex, socioeconomic status, region, and rurality. Additional methodological details are in S1 Appendix.

### Outcomes

The primary outcome was the rate of new inhaler prescriptions. The rate of new prescriptions was calculated by dividing the number of new dispensings by the total eligible persons in the population per month per 100,000. Our study assumes that a new drug dispensing is equivalent to a new clinician prescription. We identified inhaler prescriptions using drug identification numbers (DINs) associated with prescription data in ODB. DINs specific to inhalers were obtained from the Drug Product Database [22] and are listed in S2 Appendix. For each patient, we also extracted data on age group, sex, socioeconomic status, region of residence, rurality, and any prior asthma and/or COPD diagnosis. In our secondary analysis, we examined the types of inhaler devices—MDIs, DPIs, and SMIs.

### Data analysis

We defined two 36-month periods of interest: the ‘pre-pandemic’ period from April 1, 2017, to March 31, 2020, and the ‘pandemic’ period from April 1, 2020, to March 31, 2023, covering the time from the onset of the pandemic, to the end of the study, encompassing the first year of the pandemic and the later period of recovery. Although lockdown restrictions were announced before April 1, 2020, [23,24] we accounted for a likely delay in their full uptake and implementation. We compared demographic characteristics in both periods (age group, sex, rurality, income quintile, region) by conducting two-tailed chi-square tests with the null hypothesis that the proportion of people in each category did not differ in the pandemic period compared to the pre-pandemic period.

### Time series analysis

We hypothesized that there would be an immediate change (i.e., no lag) in both the trend and the slope of monthly inhaler prescription rates due to the lockdown restrictions and reduction in respiratory illnesses in the pandemic period, [25] compared to the pre-pandemic period, for all three types of inhalers. To compare monthly inhaler prescription rates during the pandemic with pre-lockdown rates, we employed an interrupted time series design without a control group, with seasonality and the hypothesized changes, along with age group, sex, income quintile, rurality, and region, as regressors.

Although segmented linear regression is commonly used in interrupted time series analyses, we selected a generalized additive model (GAM) because inhaler prescribing rates exhibited non-linear temporal patterns and strong seasonal variation. GAMs allow flexible modelling of non-linear trends through smooth functions of time, without imposing linearity within predefined segments, which may not adequately capture seasonal fluctuations in prescribing behaviour. We used GAMs to estimate the adjusted, non-linear trend separately for each category of inhaler. From these models, we derived estimated marginal means representing adjusted monthly prescription rates across study periods, facilitating comparison between pre-pandemic and pandemic intervals. The adequacy of the GAM was assessed by examining the fitted residuals (S3 Appendix).

We calculated and reported the estimated mean monthly prescription rates for each inhaler type, with 95% confidence intervals, across the two periods of interest and stratified by demographic variables.

All statistical analyses were performed using R version 4.3.2. We used the R package ‘mgcv’ to fit and evaluate the GAMs. The R package ‘emmeans’ was used to derive the estimated marginal means. [26,27] Further details on our model-building and validation processes are provided in the S3 Appendix.

## Results

### Descriptive analysis

We identified 454,931 individuals who were prescribed at least one new inhaler during our study period (Table 1), leading to a total of 750,517 new prescriptions. More individuals received new prescriptions in the pre-pandemic period (61.1%), compared to the pandemic period (38.9%). While the 65–74 year age group appeared to receive more prescriptions than other age groups during both periods, this difference was not statistically significant (p = 0.213). Similarly, we found no statistically significant differences in the number of prescriptions between males and females (p = 0.99), between income quintiles (p = 0.220), or between rural and urban populations (p = 0.99).

**Table 1:** Characteristics of persons receiving a new inhaler prescription during pre-pandemic and pandemic periods.

Characteristic	Pre-pandemic period	Pandemic period	p-value^1^
Overall, n (%)	278968(61.3)	175963(38.7)	
Age group, n (%)			
65-74 years	149838 (53.7)	98281 (55.9)	0.213
75-84 years	84886 (30.4)	50289 (28.6)	
85-94 years	39214 (14.1)	23826 (13.5)	
95 + years	5030 (1.8)	3567 (2.0)	
Sex, n (%)			
Female	156835 (56.2)	95274 (54.1)	0.990
Male	122133 (43.8)	80689 (45.9)	
Income Quintile, n (%)			
Q1 - Lowest	69895 (25.1)	40989 (23.3)	0.220
Q2	60973 (21.9)	37524 (21.3)	
Q3 - Middle	52505 (18.8)	33773 (19.2)	
Q4	47562 (17.0)	31059 (17.7)	
Q5 - Highest	48033 (17.2)	32618 (18.5)	
Rurality, n (%)			
Rural	42279 (15.2)	28233 (16.0)	0.999
Urban	236689 (84.8)	147730 (84.0)	

1. Chi-square tests.

**Fig 1:**
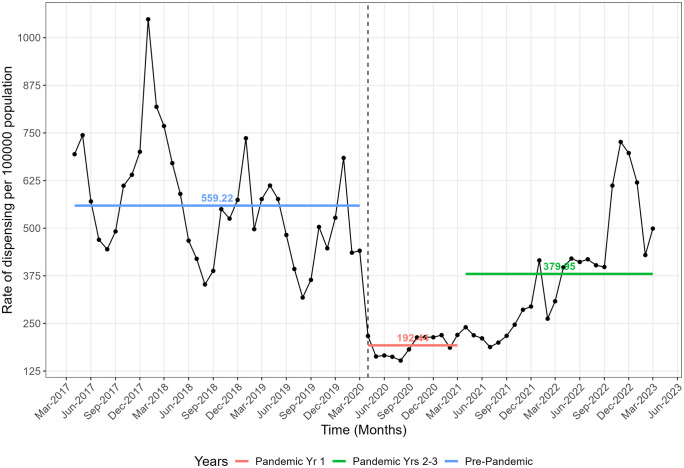
Monthly Rate of New Inhaler Prescription. **April 1, 2020, to March 31, 2023.** Pre – pandemic period: April 1, 2017, to March 31, 2021. Pandemic Yr 1: April 1, 2020, to March 31, 2021. Pandemic Yr 2-3: April 1, 2021, to March 31, 2023.

A total of 750,517 new inhalers were dispensed over the period of the study. 458,416 (60.1%) of these were in the 36 months of the pre-pandemic period, while 292,101 (38.9%) were dispensed in the 36 months of the pandemic period. The rate of new inhaler prescriptions over time is plotted in Fig 1, with the horizontal bars indicating the mean rate of prescription over a period. In the pre-pandemic period, we observed seasonal variations in the monthly rate, ranging from 1048/100,000 in January 2018–318/100,000 in August 2019, with an overall mean rate of 559 (SD = 151) for the time period. In the first year of the pandemic we observed a significant drop in mean monthly rates, reaching a low of 153/100,000 in August 2020. Within the first year, rates ranged from 153/100,000 in August 2020–220/100,000 in March 2021, with a mean rate of 192 (SD = 26). Beginning August 2021 and onwards we observed an overall increase in new inhaler prescriptions, with a peak of 612/100,000 in October 2022 which was within the range of monthly mean rates in the pre-pandemic period.

**Fig 2:**
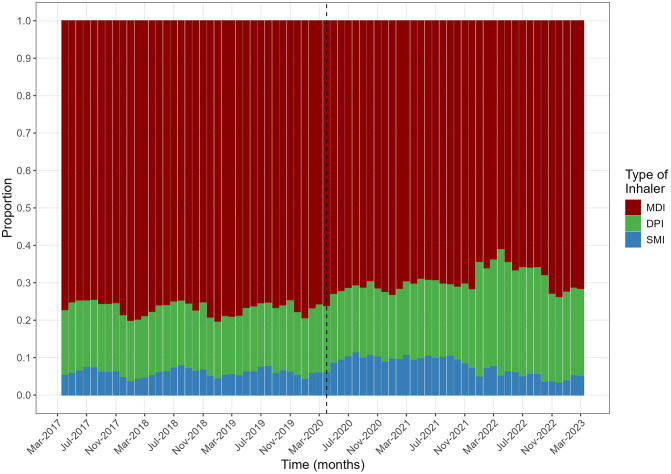
New Inhaler Prescription per type of Inhaler. April 1, 2020, to March 31, 2023.

Fig 2 shows the proportions of new prescriptions across the DPI, MDI, and SMI inhaler categories. MDIs were the predominant type of inhaler prescribed for both pre-pandemic ~77% (SD = 1.8) and pandemic ~70% (SD = 3.3) periods. The proportion of DPI prescriptions modestly increased from ~17% (SD = 1.1) to ~23% (SD = 4.6). The proportion of MDI prescriptions remained relatively constant over time until around January 2022, accounting for approximately 75% of all new prescriptions (ranging from 69% to 81% per month). Beginning in early 2022, the proportion of MDI prescriptions decreased from 75% to 68%, while DPI prescriptions saw an increase from 18% to 24%. The proportion of SMI showed a modest increase early in the pandemic, increasing from approximately 6% pre-pandemic to 7% during the first 18 months; however, this increase was not sustained, with proportions returning to levels similar to or below pre-pandemic averages by the end of follow-up.

### Time series analysis

**Fig 3:**
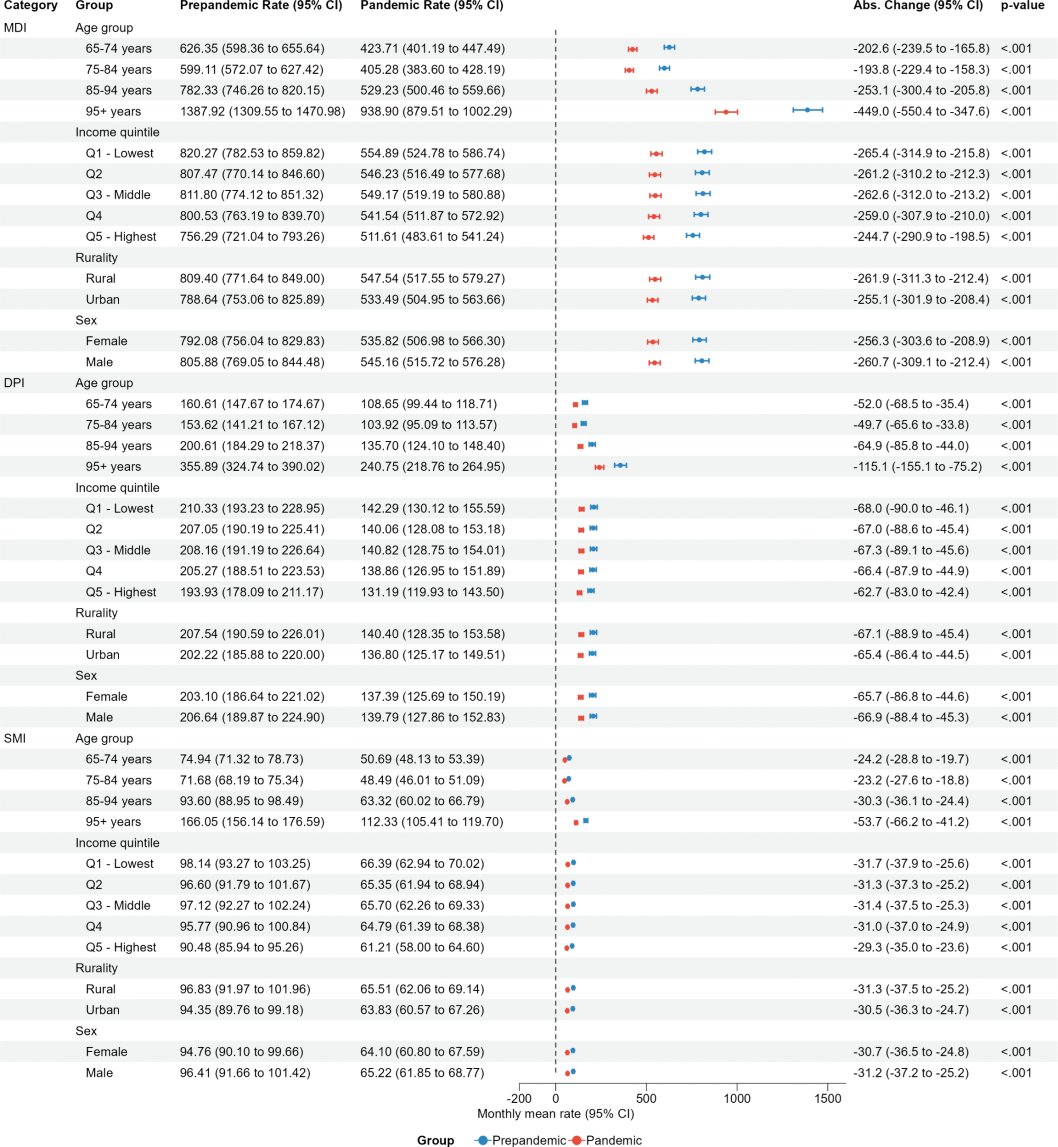
Monthly mean rate of prescription for each category (DPI, MDI, SMI) of inhaler by phase of study (pre-pandemic vs. pandemic). Pre-pandemic period: April 1, 2017, to March 31, 2020. Pandemic period: April 1, 2020, to March 31, 2023.

The results of the interrupted time series analysis using generalized additive models are shown in Fig 3 (adjusted for demographic variables) and S4 Appendix (Overall change per inhaler type). Across all inhaler types, prescribing rates declined during the pandemic compared with the pre-pandemic period, with the largest reductions observed for MDIs.

Contrasts between the pre-pandemic and pandemic periods indicate statistically significant reductions across all sociodemographic groups. For MDIs, absolute reductions ranged from −193.8 to −449.0 per 100,000 population, with the largest decline observed among individuals aged ≥95 years, who also had the highest adjusted prescription rate. Reductions were consistent across income quintiles, sex, and rurality (all p < 0.001).

For DPIs, absolute reductions ranged from −49.7 to −115.1 per 100,000, and for SMIs from −23.2 to −53.7 per 100,000 (all p < 0.001). Prescription rate patterns in pre and pandemic periods were similar across income quintiles, with consistently lower rates observed in the highest SES group, although formal statistical testing of trends across quintiles was not performed. Reductions by sex and rurality were also consistent with minor differences between groups.

In unadjusted analyses, prescription rates declined by approximately 45% during the pandemic (S4 Appendix), whereas adjusted analyses showed an approximate 33% reduction (Fig 3). Overall mean dispensing rates per 100,000 population were as follows: MDIs, 310.7 (95% CI 257.9–374.4) pre-pandemic and 172.6 (95% CI 150.5–197.9) during the pandemic; DPIs, 75.9 (95% CI 60.7–94.9) and 42.2 (95% CI 34.9–51.0); SMIs, 31.5 (95% CI 26.6–37.3) and 17.5 (95% CI 15.2–20.1), respectively.

## Discussion

This study examined the rates of new inhaler prescriptions among people aged 65 and older in Ontario from 2017 to 2023 to evaluate shifts towards more environmentally sustainable inhaler devices and to assess the impact of the COVID-19 pandemic on prescription patterns. We report mean monthly prescription rates for each inhaler category across both study phases (pre-pandemic and pandemic), overall and adjusted for age, socioeconomic status, sex, and rurality.

From April 1st, 2017, to March 31st, 2023, inhaler prescriptions exhibited seasonal variations, with a significant decline in new prescriptions observed at the onset of the pandemic in April 2020. By the end of the study period in March 2023, prescription patterns were nearing pre-pandemic levels. Additionally, there was a relative increase in the prescribing of DPIs and SMIs compared to MDIs. Although prescription rates declined across all demographic groups during the pandemic, the relative patterns of prescribing by sex, age, rurality, income, and region were broadly similar in the pre-pandemic and pandemic periods, suggesting no substantial effect modification by these variables.

The drop in prescription rates during the pandemic period may be attributed to several factors, including reduced circulation of respiratory infections, [25] limitations in access to physicians, [28–30] patients avoiding healthcare settings due to fear of COVID infection, [31,32] decreased air pollution levels, [33] and the reallocation of healthcare resources. [34] This decline in prescription rates may also be linked to the reduced incidence of outpatient visits at the onset of the pandemic. [35,36]

Prescription rates for inhaler formulations vary significantly across countries. Recent international studies report MDI prescription rates ranging from 13% to 59%, [6,37] while data from British Columbia indicate MDI rates as high as 65%. [5] This study reveals that in Ontario, MDI prescription rates are even higher, ranging from 70–80%.

Expert consensus [5,6,8,38,39] has yet to effect substantial changes in inhaler prescribing practices in Ontario. Contributing factors may include delays in knowledge translation, [40] unmeasured patient or clinical variables, [41,42] perceived patient difficulties in adopting DPIs, [43] and prescriber inertia or biases. [42,43] The stress and disruptions of the COVID-19 pandemic on both patients and prescribers may have further hindered the adoption of new prescribing practices.

This study’s strengths include a large sample size, comprehensive data sources, and recent data capturing a period affected by various factors influencing inhaler prescribing. However, there are notable limitations. First, since the dataset includes only adults aged 65 and older in Ontario, and comprehensive prescribing data for other age groups is unavailable. This limits the generalizability of our findings to younger populations, in whom inhaler prescribing patterns may differ. In addition, this restriction may introduce selection bias, as older adults have a higher burden of COPD and multimorbidity, which may influence both the frequency and type of inhaler prescribing. However, this population remains highly relevant from both a clinical and environmental perspective, as older adults account for a substantial proportion of inhaler use and healthcare utilization related to chronic respiratory diseases. Additionally, Dry Powder Inhalers (DPIs) are unsuitable for patients with insufficient respiratory effort, such as frail elderly individuals, [43] who may be disproportionately represented in this sample.

Second, while we attempted to explore the reasons for inhaler prescriptions, this was beyond the scope of the study due to limitations of the available data. For instance, we found only 75% of prescriptions were within a week of an outpatient visit, and the diagnostic codes associated with these visits may not consistently reflect the clinical reasons for prescribing inhalers, as patients may refill their prescriptions during visits for unrelated conditions, such as hypertension. Our findings should therefore be interpreted as describing population-level dispensing patterns rather than clinical decision-making. Future studies integrating richer clinical data are needed to better understand the drivers and appropriateness of inhaler prescribing.

Third, our definition of a new inhaler prescription, based on no dispensing in the prior 365 days, may capture some re-initiations, particularly in those with chronic conditions and intermittent adherence. However, this approach is commonly used in administrative data studies to approximate new use and was applied consistently across study periods, making differential bias in temporal comparisons unlikely. Additionally, as our analysis is based on dispensing data, and does not capture primary non-adherence or subsequent medication changes. As such, our findings should be interpreted as reflecting patterns of medication dispensing rather than prescribing intent.

Finally, the study does not account for the location of healthcare visits, such as emergency departments or outpatient clinics, which could also impact prescription patterns.

While our analysis primarily examines pre- and post-pandemic effects, extending the study period beyond April 2023 could yield a more comprehensive dataset for assessing future prescribing trends. Further research opportunities include: (i) quantifying changes in prescription patterns across inhaler drug classes, especially those containing inhaled corticosteroids, (ii) investigating the primary medical conditions driving inhaler prescriptions, alongside the provider and patient characteristics associated with these prescriptions, and (iii) quantifying the prescription of all environmentally preferred formulations (DPIs, SMIs) relative to non-sustainable MDIs.

## Conclusion

Our analysis shows that the COVID-19 pandemic significantly disrupted inhaler prescriptions among Ontarians aged 65 and older. Despite updated clinical guidelines advocating for a transition towards Dry Powder Inhalers (DPIs), Soft Mist Inhalers (SMIs), and lower carbon burden alternatives to mitigate environmental impact without compromising patient outcomes, Metered-Dose Inhalers (MDIs) remained predominant. The persistence reliance on MDIs indicate significant barriers in knowledge translation and suggest prescriber biases. To align prescribing practices with current evidence and sustainability goals, targeted knowledge translation initiatives are essential. Further research is needed to explore post-pandemic trends and strategies to promote the adoption of preferred inhalers.

## Supporting information

S1 Appendix Study population. (PDF)

S2 Appendix DIN List. (PDF)

S3 Appendix Model Specification. (PDF)

S4 Appendix Overall rates of inhaler prescription by category. (PDF)

S5. Appendix STROBE Statement checklist. (PDF)
