# Supplementary material for: Prescription patterns of inhaler medications from 2017 to 2023: A retrospective study using Ontario administrative healthcare data
Source: PLoS One. 2026 Jun 10;21(6):e0348119. doi: 10.1371/journal.pone.0348119 (PMC13252740; doi:10.1371/journal.pone.0348119)
Supplement: S1 Appendix — (PDF) [file pone.0348119.s001.pdf]

# S1 Appendix

## Study population

Our study included all persons registered in the Registered Persons Database (RPDB) 66 years or older who have received a inhaler prescription for the first time during the study period (April 1, 2017 to October 31, 2023). We define a person as having a new prescription if they did not have a previous inhaler prescription within the previous 365 days.

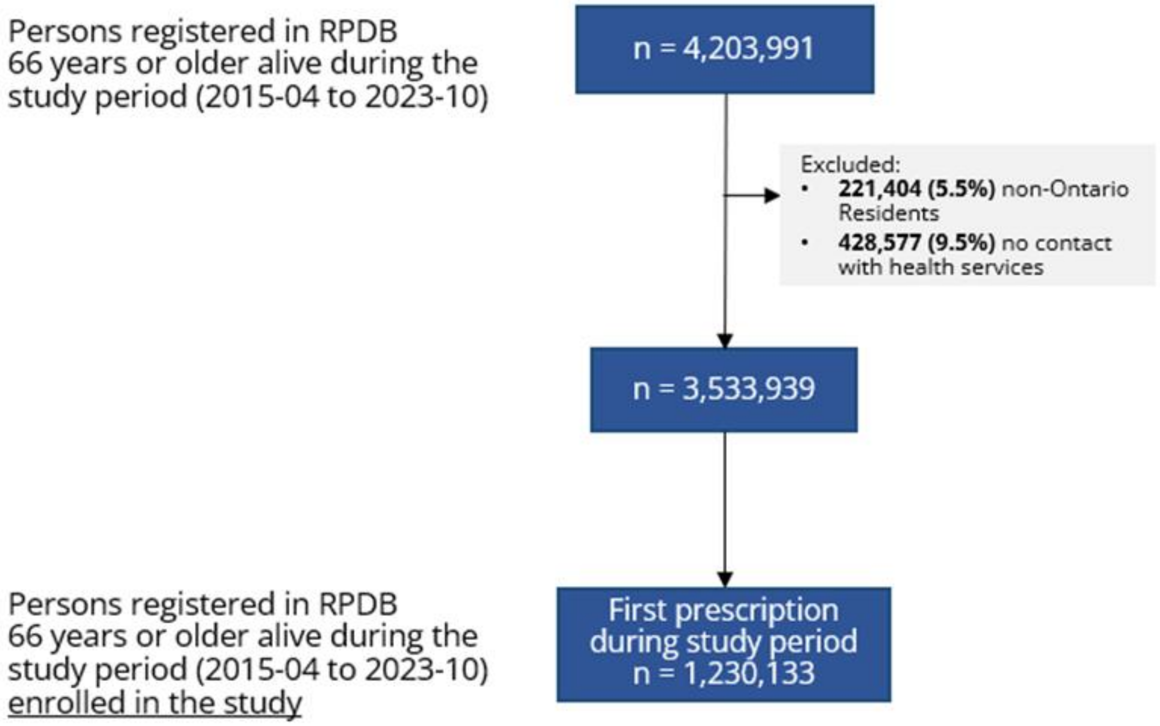


**Fig A. Flowchart of study population**

We selected adults aged 66 to 105 years who were registered in the RPDB, resided in Ontario at any point during the study period, and had valid demographic data. Prescription data were extracted from the Ontario Drug Benefit (ODB) database, which includes both new and repeat inhaler prescriptions. To identify new prescriptions, we defined them as those issued without any prior inhaler prescription in the preceding 365 days. To ensure the consistent application of the 365-day 'washout period,' we set the minimum eligibility age at 66 years, allowing for at least one year of historical data for all participants.
