# Supplementary material for: Prescription patterns of inhaler medications from 2017 to 2023: A retrospective study using Ontario administrative healthcare data
Source: PLoS One. 2026 Jun 10;21(6):e0348119. doi: 10.1371/journal.pone.0348119 (PMC13252740; doi:10.1371/journal.pone.0348119)
Supplement: S2 Appendix — (PDF) [file pone.0348119.s002.pdf]

S2 Appendix

DIN List

Table A lists all the Drug Identification Numbers (DINs) for inhalers included in the study, along with their classifications. The DINs were sourced from the Drug Product Database. We reviewed each DIN and its associated product, categorizing them by inhaler type [Metered Dose Inhalers (MDI), Dry Powder Inhalers (DPI), or Soft Mist Inhalers (SMI)] and by drug class [SABA, LABA/ICS, ICS, Other].

Table A. DIN List used in the Study.

DIN	Name	Strength	Brand Name	Type	Category
2240835	SALMETEROL (SALMETEROL XINAFOATE)	50 MCG	ADVAIR 100 DISKUS	LABA.ICS	DPI
2240835	FLUTICASONE PROPIONATE	100 MCG	ADVAIR 100 DISKUS	LABA.ICS	DPI
2245126	SALMETEROL (SALMETEROL XINAFOATE)	25 MCG	ADVAIR	LABA.ICS	MDI
2245126	FLUTICASONE PROPIONATE	125 MCG	ADVAIR	LABA.ICS	MDI
2245127	SALMETEROL (SALMETEROL XINAFOATE)	25 MCG	ADVAIR	LABA.ICS	MDI
2245127	FLUTICASONE PROPIONATE	250 MCG	ADVAIR	LABA.ICS	MDI
2240836	SALMETEROL (SALMETEROL XINAFOATE)	50 MCG	ADVAIR 250 DISKUS	LABA.ICS	DPI
2240836	FLUTICASONE PROPIONATE	250 MCG	ADVAIR 250 DISKUS	LABA.ICS	DPI
2240837	SALMETEROL (SALMETEROL XINAFOATE)	50 MCG	ADVAIR 500 DISKUS	LABA.ICS	DPI
2240837	FLUTICASONE PROPIONATE	500 MCG	ADVAIR 500 DISKUS	LABA.ICS	DPI
2467895	FLUTICASONE PROPIONATE	55 MCG	AERMONY RESPICLICK	ICS	DPI
2467909	FLUTICASONE PROPIONATE	113 MCG	AERMONY RESPICLICK	ICS	DPI
2467917	FLUTICASONE PROPIONATE	232 MCG	AERMONY RESPICLICK	ICS	DPI
2232570	SALBUTAMOL (SALBUTAMOL SULFATE)	100 MCG	AIROMIR	SABA	MDI
872334	BECLOMETHASONE DIPROPIONATE	50 MCG	ALTI-BECLOMETHASONE DIPROPIONATE INHALER 50MCG/MD	ICS	MDI
2285592	CICLESONIDE	50 MCG	ALVESCO	ICS	MDI
2285606	CICLESONIDE	100 MCG	ALVESCO	ICS	MDI
2285614	CICLESONIDE	200 MCG	ALVESCO	ICS	MDI
2418401	VILANTEROL (VILANTEROL TRIFENATATE)	25 MCG	ANORO ELLIPTA	LABA.LAMA	DPI
2418401	UMECLIDINIUM (UMECLIDINIUM BROMIDE)	62.5 MCG	ANORO ELLIPTA	LABA.LAMA	DPI
2510987	FLUTICASONE PROPIONATE	250 MCG	APO-FLUTICASONE HFA	ICS	MDI
2526557	FLUTICASONE PROPIONATE	125 MCG	APO-FLUTICASONE HFA	LABA	MDI
2528428	FLUTICASONE PROPIONATE	50 MCG	APO-FLUTICASONE HFA	LABA	MDI
2245669	SALBUTAMOL (SALBUTAMOL SULFATE)	100 MCG	APO-SALBUTAMOL HFA	SABA	MDI
2046741	SALBUTAMOL (SALBUTAMOL SULFATE)	5 MG	APO-SALVENT 5MG/ML	SABA	MDI
790419	SALBUTAMOL	100 MCG	APO-SALVENT AEM 100MCG	SABA	MDI
2474611	FLUTICASONE PROPIONATE	55 MCG	ARBESDA RESPICLICK	LABA.ICS	DPI
2474611	SALMETEROL (SALMETEROL XINAFOATE)	14 MCG	ARBESDA RESPICLICK	LABA.ICS	DPI
2474638	FLUTICASONE PROPIONATE	113 MCG	ARBESDA RESPICLICK	LABA.ICS	DPI
2474638	SALMETEROL (SALMETEROL XINAFOATE)	14 MCG	ARBESDA RESPICLICK	LABA.ICS	DPI
2474646	FLUTICASONE PROPIONATE	232 MCG	ARBESDA RESPICLICK	LABA.ICS	DPI
2474646	SALMETEROL (SALMETEROL XINAFOATE)	14 MCG	ARBESDA RESPICLICK	LABA.ICS	DPI
2446561	FLUTICASONE FUROATE	100 MCG	ARNUITY ELLIPTA	ICS	DPI
2446588	FLUTICASONE FUROATE	200 MCG	ARNUITY ELLIPTA	ICS	DPI
2243595	MOMETASONE FUROATE	200 MCG	ASMANEX TWISTHALER	ICS	DPI

2243596	MOMETASONE FUROATE	400 MCG	ASMANEX TWISTHALER	ICS	DPI
2438690	MOMETASONE FUROATE	100 MCG	ASMANEX TWISTHALER	ICS	DPI
2498685	INDACATEROL (INDACATEROL ACETATE)	150 MCG	ATECTURA BREEZHALER	LABA.ICS	DPI
2498685	MOMETASONE FUROATE	80 MCG	ATECTURA BREEZHALER	LABA.ICS	DPI
2498693	INDACATEROL (INDACATEROL ACETATE)	150 MCG	ATECTURA BREEZHALER	LABA.ICS	DPI
2498693	MOMETASONE FUROATE	320 MCG	ATECTURA BREEZHALER	LABA.ICS	DPI
2498707	INDACATEROL (INDACATEROL ACETATE)	150 MCG	ATECTURA BREEZHALER	LABA.ICS	DPI
2498707	MOMETASONE FUROATE	160 MCG	ATECTURA BREEZHALER	LABA.ICS	DPI
731439	IPRATROPIUM BROMIDE	250 MCG	ATROVENT	SAMA	MDI
2247686	IPRATROPIUM BROMIDE	20 MCG	ATROVENT HFA	SAMA	MDI
1926314	TRIAMCINOLONE ACETONIDE	200 MCG	AZMACORT	ICS	MDI
2469359	SALBUTAMOL (SALBUTAMOL SULFATE)	97 MCG	BACA RESPICLICK	SABA	DPI
2213710	BECLOMETHASONE DIPROPIONATE	100 MCG	BECLODISK - PWR INH 100MCG/BLISTER	ICS	DPI
2213729	BECLOMETHASONE DIPROPIONATE	200 MCG	BECLODISK - PWR INH 200MCG/BLISTER	ICS	DPI
828521	BECLOMETHASONE DIPROPIONATE	100 MCG	BECLODISK PWR 100MCG/BLISTER	ICS	DPI
828548	BECLOMETHASONE DIPROPIONATE	200 MCG	BECLODISK PWR 200MCG/BLISTER	ICS	DPI
897353	BECLOMETHASONE DIPROPIONATE	250 MCG	BECLOFORTE 250MCG/AEM	ICS	MDI
2215055	BECLOMETHASONE DIPROPIONATE	250 MCG	BECLOFORTE INHALER - AEM INH 250MCG/AEM	ICS	MDI
2079976	BECLOMETHASONE DIPROPIONATE	50 MCG	BECLOMETHASONE DIPROPIONATE ORAL INHALER	ICS	MDI
2216531	BECLOMETHASONE DIPROPIONATE	50 MCG	BECLOVENT - AEM 50MCG/AEM	ICS	MDI
893633	BECLOMETHASONE DIPROPIONATE	50 MCG	BECLOVENT AEM 50MCG/AEM	ICS	MDI
2215039	BECLOMETHASONE DIPROPIONATE	100 MCG	BECLOVENT ROTACAPS - INH 100MCG/CAPSULE	ICS	DPI
2215047	BECLOMETHASONE DIPROPIONATE	200 MCG	BECLOVENT ROTACAPS - INH 200MCG/CAP	ICS	DPI
1949993	BECLOMETHASONE DIPROPIONATE	100 MCG	BECLOVENT ROTACAPS 100MCG	ICS	DPI
1950002	BECLOMETHASONE DIPROPIONATE	200 MCG	BECLOVENT ROTACAPS 200MCG	ICS	DPI
541389	FENOTEROL HYDROBROMIDE	1 MG	BEROTEC 1MG/ML	SABA	MDI
2006383	FENOTEROL HYDROBROMIDE	100 MCG	BEROTEC AEM 100MCG/DOSE	SABA	MDI
371807	FENOTEROL HYDROBROMIDE	0.2 MG	BEROTEC FORTE METERED AER	SABA	MDI
2408872	VILANTEROL (VILANTEROL TRIFENATATE)	25 MCG	BREO ELLIPTA	LABA.ICS	DPI
2408872	FLUTICASONE FUROATE	100 MCG	BREO ELLIPTA	LABA.ICS	DPI
2444186	FLUTICASONE FUROATE	200 MCG	BREO ELLIPTA	LABA.ICS	DPI
2444186	VILANTEROL (VILANTEROL TRIFENATATE)	25 MCG	BREO ELLIPTA	LABA.ICS	DPI
2518058	BUDESONIDE	160 MCG	BREZTRI AEROSPHERE	LABA.ICS	MDI
2518058	GLYCOPYRRONIUM (GLYCOPYRRONIUM BROMIDE)	7.2 MCG	BREZTRI AEROSPHERE	LABA.ICS	MDI
2518058	FORMOTEROL FUMARATE DIHYDRATE	5 MCG	BREZTRI AEROSPHERE	LABA.ICS	MDI
786616	TERBUTALINE SULFATE	0.5 MG	BRICANYL TURBUHALER	SABA	DPI
2419106	IPRATROPIUM BROMIDE (IPRATROPIUM BROMIDE MONOHYDRATE)	20 MCG	COMBIVENT RESPIMAT	SABA.SAMA	SMI
2419106	SALBUTAMOL (SALBUTAMOL SULFATE)	100 MCG	COMBIVENT RESPIMAT	SABA.SAMA	SMI
2439530	ACLIDINIUM BROMIDE	400 MCG	DUAKLIR GENUAIR	LABA.LAMA	DPI
2439530	FORMOTEROL FUMARATE DIHYDRATE	12 MCG	DUAKLIR GENUAIR	LABA.LAMA	DPI

2501244	INDACATEROL (INDACATEROL ACETATE)	150 MCG	ENERZAIR BREEZHALER	LABA.LAMA.ICS	DPI
2501244	GLYCOPYRRONIUM (GLYCOPYRRONIUM BROMIDE)	50 MCG	ENERZAIR BREEZHALER	LABA.LAMA.ICS	DPI
2501244	MOMETASONE FUROATE	160 MCG	ENERZAIR BREEZHALER	LABA.LAMA.ICS	DPI
2237244	FLUTICASONE PROPIONATE	50 MCG	FLOVENT DISKUS	ICS	DPI
2237245	FLUTICASONE PROPIONATE	100 MCG	FLOVENT DISKUS	ICS	DPI
2237246	FLUTICASONE PROPIONATE	250 MCG	FLOVENT DISKUS	ICS	DPI
2237247	FLUTICASONE PROPIONATE	500 MCG	FLOVENT DISKUS	ICS	DPI
2244291	FLUTICASONE PROPIONATE	50 MCG	FLOVENT HFA	ICS	MDI
2244292	FLUTICASONE PROPIONATE	125 MCG	FLOVENT HFA	ICS	MDI
2244293	FLUTICASONE PROPIONATE	250 MCG	FLOVENT HFA	ICS	MDI
2213605	FLUTICASONE PROPIONATE	125 MCG	FLOVENT INHALERS - AEM INH-ORL 125MCG/AEM	ICS	MDI
2213613	FLUTICASONE PROPIONATE	250 MCG	FLOVENT INHALERS - AEM INH-ORL 250MCG/AEM	ICS	MDI
2213583	FLUTICASONE PROPIONATE	25 MCG	FLOVENT INHALERS - AEM INH-ORL 25MCG/AEM	ICS	MDI
2213591	FLUTICASONE PROPIONATE	50 MCG	FLOVENT INHALERS - AEM INH-ORL 50MCG/AEM	ICS	MDI
2230898	FORMOTEROL FUMARATE	12 MCG	FORADIL DRY POWDER CAPSULES FOR INHALATION	LABA	DPI
2473615	GLYCOPYRRONIUM (GLYCOPYRRONIUM BROMIDE)	8.3 MCG	GLYCOPYRRONIUM / FORMOTEROL FUMARATE DIHYDRATE PRESSURIZED INHALATION SUSPENSION	LABA.LAMA	MDI
2473615	FORMOTEROL FUMARATE DIHYDRATE	5.8 MCG	GLYCOPYRRONIUM / FORMOTEROL FUMARATE DIHYDRATE PRESSURIZED INHALATION SUSPENSION	LABA.LAMA	MDI
2423596	UMECLIDINIUM (UMECLIDINIUM BROMIDE)	62.5 MCG	INCRUSE ELLIPTA	LAMA	DPI
2441888	TIOTROPIUM (TIOTROPIUM BROMIDE MONOHYDRATE)	2.5 MCG	INSPIOLTO RESPIMAT	LABA.LAMA	SMI
2441888	OLODATEROL (OLODATEROL HYDROCHLORIDE)	2.5 MCG	INSPIOLTO RESPIMAT	LABA.LAMA	SMI
2483394	IPRATROPIUM BROMIDE (IPRATROPIUM BROMIDE MONOHYDRATE)	0.5 MG	IPRATROPIUM BROMIDE AND SALBUTAMOL SULPHATE INHALATION SOLUTION	SABA.SAMA	MDI
2483394	SALBUTAMOL (SALBUTAMOL SULFATE)	2.5 MG	IPRATROPIUM BROMIDE AND SALBUTAMOL SULPHATE INHALATION SOLUTION	SABA.SAMA	MDI
2542587	IPRATROPIUM BROMIDE	20 MCG	JAMP IPRATROPIUM HFA	SAMA	MDI
2537850	TIOTROPIUM (TIOTROPIUM BROMIDE MONOHYDRATE)	18 MCG	LUPIN-TIOTROPIUM	LAMA	DPI
2376938	INDACATEROL (INDACATEROL MALEATE)	75 MCG	ONBREZ BREEZHALER	LABA	DPI
2237224	FORMOTEROL FUMARATE DIHYDRATE	12 MCG	OXEZE TURBUHALER	LABA	DPI
2237225	FORMOTEROL FUMARATE DIHYDRATE	6 MCG	OXEZE TURBUHALER	LABA	DPI
2503158	FLUTICASONE PROPIONATE	100 MCG	PMS-FLUTICASONE	ICS	MDI
2503166	FLUTICASONE PROPIONATE	250 MCG	PMS-FLUTICASONE	ICS	MDI
2503174	FLUTICASONE PROPIONATE	500 MCG	PMS-FLUTICASONE	ICS	MDI
2503115	FLUTICASONE PROPIONATE	50 MCG	PMS-FLUTICASONE HFA	ICS	MDI
2503123	FLUTICASONE PROPIONATE	125 MCG	PMS-FLUTICASONE HFA	ICS	MDI
2503131	FLUTICASONE PROPIONATE	250 MCG	PMS-FLUTICASONE HFA	ICS	MDI
2494507	FLUTICASONE PROPIONATE	100 MCG	PMS-FLUTICASONE PROPIONATE/SALMETEROL DPI	LABA.ICS	DPI
2494507	SALMETEROL (SALMETEROL XINAFOATE)	50 MCG	PMS-FLUTICASONE PROPIONATE/SALMETEROL DPI	LABA.ICS	DPI
2494515	FLUTICASONE PROPIONATE	250 MCG	PMS-FLUTICASONE PROPIONATE/SALMETEROL DPI	LABA.ICS	DPI
2494515	SALMETEROL (SALMETEROL XINAFOATE)	50 MCG	PMS-FLUTICASONE PROPIONATE/SALMETEROL DPI	LABA.ICS	DPI
2494523	FLUTICASONE PROPIONATE	500 MCG	PMS-FLUTICASONE PROPIONATE/SALMETEROL DPI	LABA.ICS	DPI

2494523	SALMETEROL (SALMETEROL XINAFOATE)	50 MCG	PMS-FLUTICASONE PROPIONATE/SALMETEROL DPI	LABA.ICS	DPI
846414	PROCATEROL HYDROCHLORIDE HEMIHYDRATE	10 MCG	PRO-AIR AEROSOL 10MCG/AEM	LABA	MDI
851752	BUDESONIDE	200 MCG	PULMICORT TURBUHALER	ICS	DPI
851760	BUDESONIDE	400 MCG	PULMICORT TURBUHALER	ICS	DPI
852074	BUDESONIDE	100 MCG	PULMICORT TURBUHALER	ICS	DPI
2242029	BECLOMETHASONE DIPROPIONATE	50 MCG	QVAR	ICS	MDI
2242030	BECLOMETHASONE DIPROPIONATE	100 MCG	QVAR	ICS	MDI
851841	SALBUTAMOL	100 MCG	RATIO-SALBUTAMOL	SABA	MDI
860808	SALBUTAMOL (SALBUTAMOL SULFATE)	5 MG	RATIO-SALBUTAMOL	SABA	MDI
1986864	SALBUTAMOL (SALBUTAMOL SULFATE)	1 MG	RATIO-SALBUTAMOL	SABA	MDI
2239365	SALBUTAMOL (SALBUTAMOL SULFATE)	0.5 MG	RATIO-SALBUTAMOL	SABA	MDI
2239366	SALBUTAMOL (SALBUTAMOL SULFATE)	2 MG	RATIO-SALBUTAMOL	SABA	MDI
2244914	SALBUTAMOL (SALBUTAMOL SULFATE)	100 MCG	RATIO-SALBUTAMOL HFA	SABA	MDI
2419858	SALBUTAMOL (SALBUTAMOL SULFATE)	100 MCG	SALBUTAMOL HFA	SABA	MDI
2394936	GLYCOPYRRONIUM (GLYCOPYRRONIUM BROMIDE)	50 MCG	SEEBRI BREEZHALER	LAMA	DPI
2211742	SALMETEROL (SALMETEROL XINAFOATE)	25 MCG	SEREVENT (25MCG/ACTUATION)	LABA	MDI
2214261	SALMETEROL (SALMETEROL XINAFOATE)	50 MCG	SEREVENT DISKHALER DISK (50MCG/DOSE)	LABA	DPI
2231129	SALMETEROL (SALMETEROL XINAFOATE)	50 MCG	SEREVENT DISKUS (50MCG/DOSE)	LABA	DPI
2136139	SALMETEROL (SALMETEROL XINAFOATE)	25 MCG	SEREVENT- AEM 25MCG/AEM	LABA	MDI
2136147	SALMETEROL (SALMETEROL XINAFOATE)	50 MCG	SEREVENT-PWR 50MCG/BLISTER PACK	LABA	DPI
2246793	TIOTROPIUM (TIOTROPIUM BROMIDE MONOHYDRATE)	18 MCG	SPIRIVA	LAMA	SMI
2435381	TIOTROPIUM (TIOTROPIUM BROMIDE MONOHYDRATE)	2.5 MCG	SPIRIVA RESPIMAT	LAMA	SMI
2407868	OLODATEROL (OLODATEROL HYDROCHLORIDE)	2.5 MCG	STRIVERDI RESPIMAT	LABA	SMI
2245385	BUDESONIDE	100 MCG	SYMBICORT 100 TURBUHALER	LABA.ICS	DPI
2245385	FORMOTEROL FUMARATE DIHYDRATE	6 MCG	SYMBICORT 100 TURBUHALER	LABA.ICS	DPI
2245386	BUDESONIDE	200 MCG	SYMBICORT 200 TURBUHALER	LABA.ICS	DPI
2245386	FORMOTEROL FUMARATE DIHYDRATE	6 MCG	SYMBICORT 200 TURBUHALER	LABA.ICS	DPI
2248218	FORMOTEROL FUMARATE DIHYDRATE	12 MCG	SYMBICORT FORTE TURBUHALER	LABA.ICS	DPI
2248218	BUDESONIDE	400 MCG	SYMBICORT FORTE TURBUHALER	LABA.ICS	DPI
2465949	BUDESONIDE	0.125 MG	TEVA-BUDESONIDE	ICS	MDI
2465957	BUDESONIDE	0.5 MG	TEVA-BUDESONIDE	ICS	MDI
2535416	BUDESONIDE	0.25 MG	TEVA-BUDESONIDE	ICS	MDI
2326450	SALBUTAMOL (SALBUTAMOL SULFATE)	100 MCG	TEVA-SALBUTAMOL HFA	SABA	MDI
2474522	FLUTICASONE FUROATE	100 MCG	TRELEGY ELLIPTA	LABA.LAMA.ICS	DPI
2474522	UMECLIDINIUM (UMECLIDINIUM BROMIDE)	62.5 MCG	TRELEGY ELLIPTA	LABA.LAMA.ICS	DPI
2474522	VILANTEROL (VILANTEROL TRIFENATATE)	25 MCG	TRELEGY ELLIPTA	LABA.LAMA.ICS	DPI
2515776	FLUTICASONE FUROATE	200 MCG	TRELEGY ELLIPTA	ICS	DPI
2515776	UMECLIDINIUM (UMECLIDINIUM BROMIDE)	62.5 MCG	TRELEGY ELLIPTA	ICS	DPI
2515776	VILANTEROL (VILANTEROL TRIFENATATE)	25 MCG	TRELEGY ELLIPTA	ICS	DPI

2409720	ACLIDINIUM BROMIDE	400 MCG	TUDORZA GENUAIR	LAMA	DPI
2418282	GLYCOPYRRONIUM (GLYCOPYRRONIUM BROMIDE)	50 MCG	ULTIBRO BREEZHALER	LABA.LAMA	DPI
2418282	INDACATEROL (INDACATEROL MALEATE)	110 MCG	ULTIBRO BREEZHALER	LABA.LAMA	DPI
374407	BECLOMETHASONE DIPROPIONATE	50 MCG	VANCERIL AEM 50MCG	ICS	MDI
2214997	SALBUTAMOL (SALBUTAMOL SULFATE)	200 MCG	VENTODISK 200MCG	SABA	DPI
2215004	SALBUTAMOL (SALBUTAMOL SULFATE)	400 MCG	VENTODISK 400MCG	SABA	DPI
832766	SALBUTAMOL (SALBUTAMOL SULFATE)	200 MCG	VENTODISK PWR 200 MCG/BLISTER	SABA	DPI
832758	SALBUTAMOL (SALBUTAMOL SULFATE)	400 MCG	VENTODISK PWR 400 MCG/BLISTER	SABA	DPI
2243115	SALBUTAMOL (SALBUTAMOL SULFATE)	200 MCG	VENTOLIN DISKUS	SABA	DPI
2241497	SALBUTAMOL (SALBUTAMOL SULFATE)	100 MCG	VENTOLIN HFA	SABA	MDI
2213478	SALBUTAMOL	100 MCG	VENTOLIN INHALER	SABA	MDI
867179	SALBUTAMOL	100 MCG	VENTOLIN INHALER 100MCG/AEM	SABA	MDI
2212315	SALBUTAMOL (SALBUTAMOL SULFATE)	200 MCG	VENTOLIN ROTACAPS	SABA	DPI
2212323	SALBUTAMOL (SALBUTAMOL SULFATE)	400 MCG	VENTOLIN ROTACAPS	SABA	DPI
1938878	SALBUTAMOL (SALBUTAMOL SULFATE)	200 MCG	VENTOLIN ROTACAPS 200 MCG	SABA	DPI
1938851	SALBUTAMOL (SALBUTAMOL SULFATE)	400 MCG	VENTOLIN ROTACAPS 400 MCG	SABA	DPI
2495597	FLUTICASONE PROPIONATE	100 MCG	WIXELA INHUB	LABA.ICS	DPI
2495597	SALMETEROL (SALMETEROL XINAFOATE)	50 MCG	WIXELA INHUB	LABA.ICS	DPI
2495600	FLUTICASONE PROPIONATE	250 MCG	WIXELA INHUB	LABA.ICS	DPI
2495600	SALMETEROL (SALMETEROL XINAFOATE)	50 MCG	WIXELA INHUB	LABA.ICS	DPI
2495619	FLUTICASONE PROPIONATE	500 MCG	WIXELA INHUB	LABA.ICS	DPI
2495619	SALMETEROL (SALMETEROL XINAFOATE)	50 MCG	WIXELA INHUB	LABA.ICS	DPI
2361744	MOMETASONE FUROATE	50 MCG	ZENHALE	LABA.ICS	MDI
2361744	FORMOTEROL FUMARATE DIHYDRATE	5 MCG	ZENHALE	LABA.ICS	MDI
2361752	MOMETASONE FUROATE	100 MCG	ZENHALE	LABA.ICS	MDI
2361752	FORMOTEROL FUMARATE DIHYDRATE	5 MCG	ZENHALE	LABA.ICS	MDI
2361760	MOMETASONE FUROATE	200 MCG	ZENHALE	LABA.ICS	MDI
2361760	FORMOTEROL FUMARATE DIHYDRATE	5 MCG	ZENHALE	LABA.ICS	MDI
874086	SALBUTAMOL	100 MCG	NOVO-SALMOL INHALER 100MCG/AEM	SABA	MDI
2069571	SALBUTAMOL (SALBUTAMOL SULFATE)	5 MG	PMS-SALBUTAMOL	SABA	MDI
2154412	SALBUTAMOL (SALBUTAMOL SULFATE)	5 MG	SANDOZ SALBUTAMOL	SABA	MDI
2208229	SALBUTAMOL (SALBUTAMOL SULFATE)	1 MG	PMS-SALBUTAMOL	SABA	MDI
2208237	SALBUTAMOL (SALBUTAMOL SULFATE)	2 MG	PMS-SALBUTAMOL	SABA	MDI
2174758	FLUTICASONE PROPIONATE	50 MCG	FLOVENT INHALERS-AEM INH-ORL 50MCG/ACTUATION	ICS	ICS
2174766	FLUTICASONE PROPIONATE	125 MCG	FLOVENT INHALERS-AEM INH-ORL125MCG/ACTUATION	ICS	ICS
2174774	FLUTICASONE PROPIONATE	250 MCG	FLOVENT INHALERS-AEM INH-ORL250MCG/ACTUATION	ICS	ICS
