# Supplementary material for: Prescription patterns of inhaler medications from 2017 to 2023: A retrospective study using Ontario administrative healthcare data
Source: PLoS One. 2026 Jun 10;21(6):e0348119. doi: 10.1371/journal.pone.0348119 (PMC13252740; doi:10.1371/journal.pone.0348119)
Supplement: S3 Appendix — (PDF) [file pone.0348119.s003.pdf]

S3 Appendix

Model Specification

Data and Variables

We analyzed the number of prescriptions in each category per month for each study phase. Using DPIs as the reference group, we modeled the number of prescriptions per month as a Poisson variable with a population-based offset. Study phases were represented by a binary indicator variable (0 or 1), while a second variable captured the post-intervention trend by taking the value 0 during the pre-pandemic phase and counting months since intervention during the pandemic phase.

Interrupted Time Series Framework

An interrupted time series design is generally analyzed using a segmented regression approach, as shown in the equation below[49]:

$$Y_t = \beta_0 + \beta_1 T + \delta_1 I_t + \delta_2 T I_t + e_t$$

Where:

- Y_t represents the outcome at time T,
- β_0 represents the baseline rate at time T = 0 (prior to the intervention),
- β_1 is interpreted as the change in the outcome per one unit change in time in the pre-intervention period,
- δ_1 represents the coefficient for the indicator variable showing the presence or absence of the intervention, interpreted as the level change in outcome associated with the introduction of the intervention and
- δ_2 represents the coefficient of the indicator variable showing time since the intervention, interpreted as the slope change in the outcome after introducing the intervention.

Generalized Additive Model (GAM) Approach

To account for seasonality, autocorrelation, and non-linear trends, we extended the ITS design using GAMs. GAMs model the outcome as a linear function of smooth predictor terms, using smoothing splines that join piecewise polynomials at data "knots." The optimal number of smoothing functions is determined by minimizing the penalized sum of squares, balancing adequate non-linearity and overfitting via the parameter λ.[49,50].

Our model can be represented as:

$$Yt = \beta_0 + s_1(T) ++ \delta_1 I_t + \delta_2 T I_t + e_t$$

where s_1 is the smoothing function of time.

Model Development

We used a cyclic cubic spline as the smoothing function in the model to account for seasonality and autocorrelation. Starting with the default number of knots (20) in the model, we progressively increased the number until the autocorrelation in the residuals at 24 lags was sufficiently controlled, while also ensuring the model fit the data sufficiently well. This approach allowed us to obtain adjusted ratios by controlling for socioeconomic characteristics.

We used the default methods provided in the mgcv package to estimate the best fit and the Restricted Maximum Likelihood (REML) option to estimate coefficients. The 'game.check' and 'plot' functions were used to examine the model's fit. The final model used 56 knots, including two boundary knots, with separate smoothing functions for each type of inhaler and linear covariates (age group, sex, rurality, SES and region). Autocorrelation in the residuals was limited to an absolute value of 0.43 for up to 24 lags. The goodness-of-fit was estimated by examining the model plots, which are reproduced below.

Model Diagnostic plots

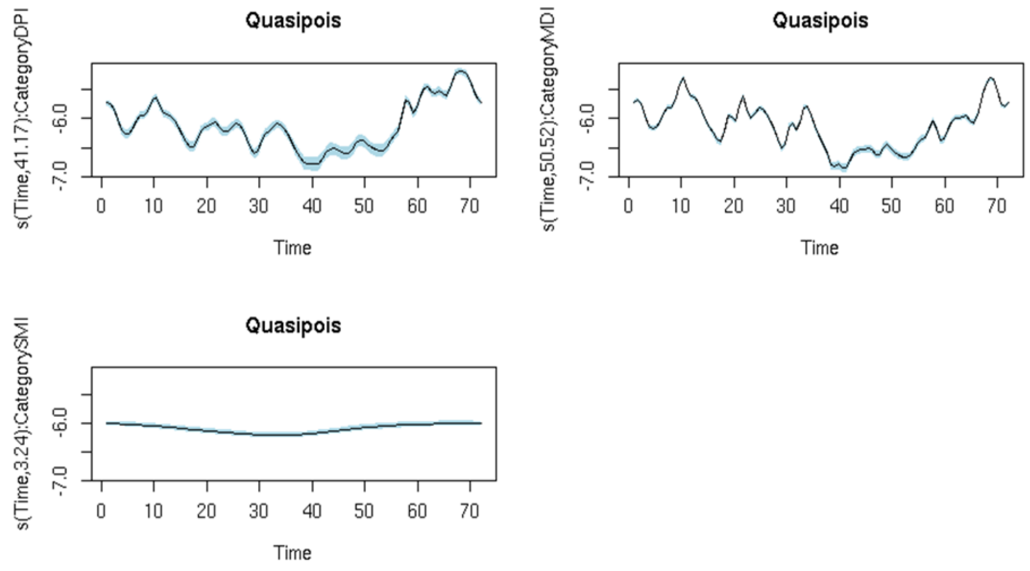

Fig A: Fitted values of the gam model

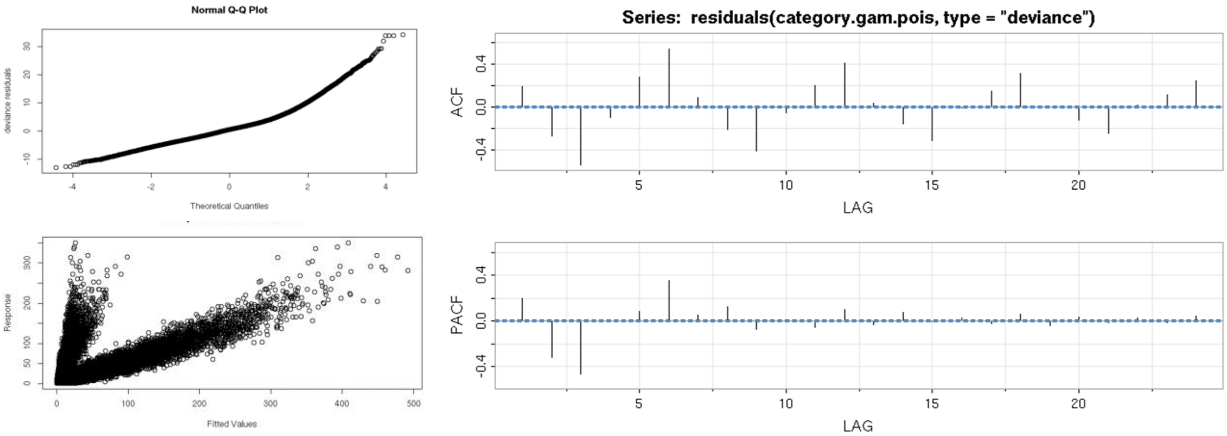

Fig B: QQ plot of model residuals, response vs. fitted value plot and autocorrelation plot (ACF, PACF) of model residuals. These suggest a good fit for the model, with autocorrelation controlled to a large extent.

The model achieved an adjusted R-squared of 20% and explained approximately 45% of the observed deviance. The effective degrees of freedom for DPIs, MDIs, and SMIs were 41.17, 50.52, and 3.24, respectively, which is reflected in the variability of the fitted value plots.
