# Supplementary material for: Prescription patterns of inhaler medications from 2017 to 2023: A retrospective study using Ontario administrative healthcare data
Source: PLoS One. 2026 Jun 10;21(6):e0348119. doi: 10.1371/journal.pone.0348119 (PMC13252740; doi:10.1371/journal.pone.0348119)
Supplement: S4 Appendix — (PDF) [file pone.0348119.s004.pdf]

S4 Appendix.

Overall rates of inhaler prescription by category

Group	Prepandemic Rate (95% CI)	Pandemic Rate (95% CI)
Catgeory of inhaler		
MDI	310.74 (257.89, 374.42)	172.6 (150.5, 197.94)
DPI	75.9 (60.71, 94.9)	42.16 (34.88, 50.96)
SMI	31.51 (26.63, 37.28)	17.5 (15.22, 20.12)

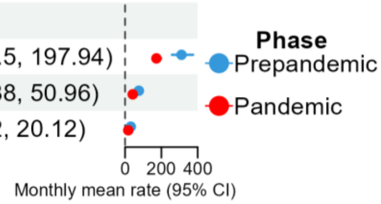

Fig A. Overall rates of inhaler prescription by category, before and during the pandemic.

Overall mean prescription rates for MDIs were 310.74 (95% CI: 257.89, 374.42) and 172.6 (95% CI: 150.5, 197.94) per 100,000 population in the pre-pandemic and pandemic periods, respectively. Overall DPI prescription rates of 75.9 (95% CI: 60.71, 94.9) and 42.16 (95% CI: 34.88, 50.96) and SMI prescription rates of 31.51 (95% CI: 26.63, 37.28) and 17.5 (95% CI: 15.22, 20.12).
